# CAT-DTI: cross-attention and Transformer network with domain adaptation for drug-target interaction prediction

**DOI:** 10.1186/s12859-024-05753-2

**Published:** 2024-04-02

**Authors:** Xiaoting Zeng, Weilin Chen, Baiying Lei

**Affiliations:** 1 School of Computer and Software, Shenzhen University, Shenzhen, 518060 China; 2 Marshall Laboratory of Biomedical Engineering, Shenzhen University Medical School, Shenzhen University, Shenzhen, 518055 China; 3 School of Biomedical Engineering, Shenzhen University, Shenzhen, 518055 China

**Keywords:** Drug-target interaction, Transformer, Cross-attention, Domain adaptation

## Abstract

Accurate and efficient prediction of drug-target interaction (DTI) is critical to advance drug development and reduce the cost of drug discovery. Recently, the employment of deep learning methods has enhanced DTI prediction precision and efficacy, but it still encounters several challenges. The first challenge lies in the efficient learning of drug and protein feature representations alongside their interaction features to enhance DTI prediction. Another important challenge is to improve the generalization capability of the DTI model within real-world scenarios. To address these challenges, we propose CAT-DTI, a model based on cross-attention and Transformer, possessing domain adaptation capability. CAT-DTI effectively captures the drug-target interactions while adapting to out-of-distribution data. Specifically, we use a convolution neural network combined with a Transformer to encode the distance relationship between amino acids within protein sequences and employ a cross-attention module to capture the drug-target interaction features. Generalization to new DTI prediction scenarios is achieved by leveraging a conditional domain adversarial network, aligning DTI representations under diverse distributions. Experimental results within in-domain and cross-domain scenarios demonstrate that CAT-DTI model overall improves DTI prediction performance compared with previous methods.

## Introduction

Drug discovery is highly valued in the current biomedical field [1]. In drug discovery, verifying whether a drug interacts with a certain target is a key step in proving drug effectiveness [2]. In vitro screening experiments are feasible but labor-intensive, expensive and time-consuming [3]. The utilization of computerized screening for potential DTI candidates has been substantiated as an effective strategy to aid biologists in the identification of genuine DTIs through wet-lab experiments [4]. Therefore, computer-aided DTI prediction has aroused great interest and received increasing attention.

Existing DTI prediction methods can generally be divided into three categories: ligand-based, structure-based and machine learning-based [5]. Traditional structure-based and ligand-based in silico virtual screening methods have gained increased attention owing to the demonstrated relative effectiveness [6–8]. However, these traditional methods have significant limitations. For example, the widely adopted molecular docking method is relatively inefficient, sometimes slow due to the huge amount of computation and the scoring function has relatively low accuracy [9]. In short, ligand-based methods face limited application scope and performance challenge due to the limited number of known ligands for some proteins. In addition, structural-based methods are limited by the lack of three-dimensional structures and ligand information for the majority of protein, thereby constraining the development.

Traditional machine learning models such as support vector machine (SVM) [10] and random forest (RF) [11] are also used for DTI prediction [12]. For example, Faulon et al. [13] used molecular features and reaction features as input to the SVM kernel function for DTI prediction. Wang et al. [14] used the features extracted by the Boruta algorithm as the input of the RF algorithm for DTI prediction. However, although these methods are simple and effective, the performance is far from satisfactory.

Recently, as an important branch of machine learning, deep learning has also made significant progress in DTI prediction. In the early days, researchers used hand-crafted descriptors of drugs and proteins to make predictions through a fully connected neural network [15]. Subsequently, Lee et al. proposed DeepConv-DTI [16], which used convolution neural network (CNN) to extract protein features, employed the extended connectivity fingerprints (ECFP) algorithm [17] to calculate drug features, and predicted DTI through fully connected network (FCN). However, the interaction characteristics of drug-protein pairs are ignored. Furthermore, the adoption of advanced feature extraction techniques, such as the DynCNN module in SAG-DTA [18] and DrugVQA [19], highlight the continuous efforts to optimize feature extraction methods.

Over the past few years, graph neural network (GNN) have demonstrated excellent predictive performance in addressing key prediction challenges in the field of bioinformatics by utilizing the powerful feature representation learning capability [20–22]. To extract the topological information of drugs, Nguyen et al. designed GraphDTA [23] based on GNN, treating drugs as molecular graphs, using GNN and CNN to extract drug and protein features respectively to predict the affinity of drugs and targets. Despite the use of stronger feature extraction modules, the important fact that the interactions between molecules are mainly focused on the relevant substructures of drugs and proteins is ignored [24]. Furthermore, a simply connection of drug and protein features fails to capture the complex interactions between them.

In order to more accurately model the interactions between drugs and proteins, some studies have introduced the attention mechanism into DTI prediction [25]. Nowadays, attention mechanisms have been widely used for revealing the contribution of different components of a drug or target on interaction [19] and describing interactions between targets and drugs [26]. HyperAttentionDTI [27] assigned attention vectors to each atom and amino acid on the basis of CNN to enhance feature expression. Although this method considered the interactions representation between drugs and proteins, the limited receptive field of CNN limits the ability to capture global dependencies.

Inspired by the powerful ability of Transformer [28] to capture features between two sequences, Chen et al. proposed TransformerCPI [29], using Transformer to predict DTI from the SMILES of drugs and protein amino acid sequences. Huang et al. proposed MolTrans [30], which applied Transformer to extract features from the substructure of drug and protein sequence and combined them into interaction map for compound-protein interaction prediction. However, this method mainly focuses on the interaction features between drugs and protein substructures, while ignoring the importance of utilizing original feature information.

Due to the wide scope and complexity of the chemical and genomic fields, DTI prediction often faces great challenges in real-world scenarios. Recently, Bai et al. proposed the DrugBAN [31] using bilinear attention to capture the local interaction representation of drug and target for DTI prediction. In cross-domain prediction tasks, the conditional adversarial domain adaptation method is introduced to transfer source domain knowledge to the target domain and demonstrate excellent cross-domain generalization capability.

In order to cope with the problem that many models [16, 23, 27, 31] cannot fully capture global context information while retaining local features when processing global and local information, resulting in the inability to extract sufficiently effective feature information. In this work, we propose a model named CAT-DTI, whose protein features are extracted by a protein feature encoder combining CNN and Transformer, which fully considers global context information while capturing local features of protein sequences. Besides, attention-based methods [23, 32–34] generally focus more on extracting internal features of drugs and targets, but rarely introduce attention to mine DTI representations. With the aim of better preserving the internal features of drugs and proteins while deeply exploring the interaction information between them, we input the extracted drug and protein features into the cross-attention module for feature fusion. We also notice that the training of the model in a specific domain is mainly aimed at the distribution of the domain and the drug-target pairs to be predicted in practical applications may have a different distribution from the training data, resulting in the inability to directly transfer the existing knowledge to new scenarios. Therefore, in cross-domain tasks, we employ conditional domain adversarial network (CDAN) to better understand and predict DTI in domains that are distributed differently from the training data, thus enhancing the cross-domain generalization ability.

Our contributions are summarized in the following points. First, we propose a deep learning model named CAT-DTI, which uses GCN and CNN combined with Transformer to extract feature maps of drugs and proteins, respectively. Second, we employ cross-attention module that fuse drug and protein features, effectively capture and process the interaction features between drugs and proteins while retaining the internal feature information. For the cross-domain prediction task, we adopt the CDAN to enable the model to adapt and cope with the characteristics of new domains, improve the performance of CAT-DTI in cross domain DTI prediction tasks and enhance the generalization performance and practical value of CAT-DTI. Compared with other baseline methods, CAT-DTI achieves generally better predictive performance on three public datasets.

## Methods

**Fig. 1:**
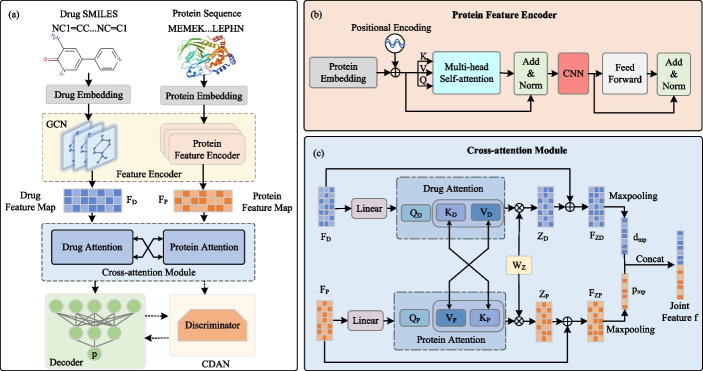
Framework of the proposed CAT-DTI. **a** Overview of CAT-DTI framework. **b** Details of the protein feature encoder. **c** Processes of cross-attention

An overview of CAT-DTI framework is illustrated in Fig. 1a. Given drug SMILES and protein amino acid sequences as input, the protein and drug embeddings are generated. Drug embedding is input into GCN to extract feature representations of drug molecules (i.e., drug feature map $F_D$). The protein embedding is passed to the protein feature encoder as shown in Fig. 1b, which combines the CNN and Transformer to extract the protein feature map $F_P$, capturing local features and global context information in the protein sequence simultaneously. Next, the cross-attention module interacts protein and drug features for feature fusion to capture the interaction relationship between drugs and targets, as shown in Fig. 1c. Specifically, we swap the key and value of protein attention with those of drug attention. After obtaining the feature maps, the original features are integrated to construct the final features for both drugs and proteins. Through max-pooling and concatenation, the joint feature *f* for drug and protein target is produced and input into the decoder to predict DTI. To enhance the generalization performance of CAT-DTI in real-world scenarios for novel drug-target pairs, we integrate the domain adaptation module CDAN into the framework, which is employed to adapt the representations of drugs and proteins, thereby facilitating effective alignment between source and target domain distributions.

### GCN for drug molecular graph

Regarding the drug feature extraction process, we transform drug SMILES into a corresponding 2D molecular graph. To capture the node information within the graph, we first initialize each atom node. Each atom is denoted by a 74-dimensional integer vector that encapsulates eight distinct attributes, including the atom type, the atom degree, the number of implicit Hs, the formal charge, the number of radical electrons, the atom hybridization, the number of total Hs and whether the atom is aromatic.

The drug feature encoder transmits and aggregates information on the drug molecular structure through a three-layer GCN, thereby achieving extraction and representation of drug feature. In each layer of GCN operation, each row of the drug representation represents an aggregated representation of adjacent atomic nodes in the drug molecule. Each GCN layer uses the information of neighboring atomic nodes to update the feature representation of each atomic node, allowing the model to effectively capture the correlation information between neighboring atomic nodes. We retain node-level drug representations for subsequent explicit learning of interactions with protein fragments. We set the maximum number of nodes in the graph to be $m_d$. Therefore, the node feature matrix of each graph is denoted as $M_d\in \mathbb {R}^{m_d\times 74}$. Furthermore, we employ a simple linear transformation to establish $F_{d}=M_{d}W_{o}^{\top }$, resulting in a real-valued dense matrix $F_d\in \mathbb {R}^{m_d\times D_d}$ as input features, where $D_d$ is the drug embedding dimension. Finally, we obtain the drug feature map $F_D\in \mathbb {R}^{m_d\times D_d}$ through the drug feature encoder, which can be expressed as:

1 $$\begin{aligned} H_d^{i+1}=\sigma (\text {GCN}(\widetilde{A},W_{gcn}^i,b_{gcn}^i,H_d^i)), \end{aligned}$$

where $W_{gcn}^i$ and $b_{gcn}^i$ are the weight matrices and bias vector of the *i*-th layer of GCN. $\tilde{A}$ is the adjacency matrix with added self-connection. $H_{d}^i$ denotes the hidden node representation of layer *i* with $H_{d}^{0}=F_{d}$.

### Feature encoder for protein

To enhance protein sequence feature representation and capture long-distance relationship between sequence tags, we introduce a protein feature encoder that combines CNN and Transformer. Traditional CNN may struggle with long sequences due to the limited local receptive fields, so we combine the global attention mechanism of Transformer to capture long-distance dependence in protein sequences. By fusing the local perception capabilitiy of CNN and the global attention mechanism of Transformer, our model simultaneously considers local features and global context information in protein sequences, thereby extracting more effective protein features.

It is worth noting that before the feed forward layer of Transformer, we add 1D CNN to process local information. By sliding the convolution kernel on the protein sequence, we captured the local pattern and substructure of the protein. Combined with the advantages of Transformer in handling long-range dependencies, our model achieves the fusion of local and global information in the protein feature encoding process, which is beneficial to enhance the representation of protein sequence features. In our work, a three-layer protein feature encoder is used to capture protein features, as shown in Fig. 1b, where each layer includes a multi-head self-attention, CNN and a feed-forward neural network. Specifically, the protein sequence is input to the protein feature encoder with the feature matrix $F_p\in \mathbb {R}^{l_p\times D_p}$, where $l_p$ is the length of the protein sequence and $D_p$ is the protein embedding dimension. The matrices $Q\in \mathbb {R}^{l_p\times D_p}$, $K\in \mathbb {R}^{l_p\times D_p}$ and $V\in \mathbb {R}^{l_p\times D_p}$ in different feature spaces based on the feature matrix $F_p$ are generated by the linear layer as follows:

2 $$\begin{aligned} {\left\{ \begin{array}{ll}Q=F_p\cdot W_Q+b_Q\\ K=F_p\cdot W_K+b_K,\\ V=F_p\cdot W_V+b_V\end{array}\right. } \end{aligned}$$

where $W_Q\in \mathbb {R}^{D_p\times D_p}$, $W_K\in \mathbb {R}^{D_p\times D_p}$, $W_V\in \mathbb {R}^{D_p\times D_p}$ are learnable parameter weights. $b_Q$, $b_K$ and $b_V$ are bias vectors. Given *Q*, *K* and *V* matrices, the self-attention layer computes the attention weights as follows:

3 $$\begin{aligned} \text {Attention}(Q,K,V)=\text {Softmax}(\frac{Q\cdot K^\top }{\sqrt{d_k}})V, \end{aligned}$$

where $d_k$ is the dimension of *K*. The output $X_M$ of the multi-head attention layer is generated as follows:

4 $$\begin{aligned} \begin{aligned} X_M&=\text {MutiHead}(Q,K,V)=\text {Concat}(\text {Attention}(Q,K,V))W_M+b_M, \end{aligned} \end{aligned}$$

where $W_M\in \mathbb {R}^{D_p\times D_p}$ is the learnable weight matrix and $b_M$ is the bias vector.

The multi-head attention layer extracts information from diverse representation subspace, enhancing model robustness. Therefore, long-range relationships between amino acids spanning the entire sequence can be learned with self-attention weights. Additionally, the first ADD & Norm layer implements a residual connection with original protein feature matrix $F_p$ and then follow by normalization, expressed as follows:

5 $$\begin{aligned} X_{AN}=\text {LayerNorm}(F_{p}+X_{M}), \end{aligned}$$

Subsequently, a three-layer CNN is inserted after the first ADD & Norm layer to extract local feature in the protein sequence:

6 $$\begin{aligned} X_{CNN}=\textrm{CNN}\left( X_{AN}\right) , \end{aligned}$$

After the second ADD & Norm layer, we derive the protein feature map $F_{P}\in \mathbb {R}^{l_p \times D_p}$ as follows:

7 $$\begin{aligned} F_P=\text {LayerNorm}(X_{CNN}+X_{AN}), \end{aligned}$$

### Cross-attention module

After obtaining the feature maps for drugs and proteins through the feature encoder, we introduce a cross-attention module to effectively model the interaction between drugs and proteins, thereby capturing enhanced representations of their interaction and provides more reliable feature representation for DTI prediction. By performing two-way information interaction between the key and value of protein attention and the key and value of drug attention, the information exchange and association between drug and protein is realized, thus capturing the interaction features between drug and protein target. In this process, protein features can adjust their own expression by attention weights of drug features, and vice versa. Such an interaction and adjustment mechanism enable the cross-attention module to promote information flow across feature maps, effectively fuse drug and protein features, and extract more comprehensive DTI feature representation. The cross-attention module is depicted in Fig. 1c and primarily consists of drug and protein attention.

In this section, we set $D_e=D_d=D_p$. For drugs, the drug feature map $F_D$ is passed through the linear layer to calculate the drug query vector $Q_D^i\in \mathbb {R}^{m_d\times d_{head}}$, and then the protein feature map $F_P$ is obtained through a linear layer, which is further calculated as the drug key vector $K_D^i\in \mathbb {R}^{l{_p\times d}_\textit{head}}$ and value vector $V_D^i\in \mathbb {R}^{l{_p\times d}_\textit{head}}$. The query, key and value for the drug are obtained as follows:

8 $$\begin{aligned} {\left\{ \begin{array}{ll}Q_D^i=F_D\cdot W_q^i\\ K_D^i=F_P\cdot W_k^i ,\\ V_D^i=F_P\cdot W_\nu ^i\end{array}\right. } \end{aligned}$$

where $$\begin{aligned}W_q^i, W_k^i, W_\nu ^i\in \mathbb {R}^{D_e\times d_{head}}\end{aligned}$$ are different weight matrices in the linear layer and $d_{head}=D_{e}/heads$ is the channel dimension. $i=1,2,\cdots ,heads$, where *heads* are the number of attention heads.

Protein attention follows a process similar to drug attention. The protein feature map $F_P$ is input into the linear layer to calculate the protein query vector $Q_P^i\in \mathbb {R}^{l{_p\times d}_\textit{head}}$, and then the drug feature map is taken to generate the protein key vector $K_P^i\in \mathbb {R}^{m{_d\times d}_\textit{head}}$ and protein value vector $V_P^i\in \mathbb {R}^{m{_d\times d}_\textit{head}}$. The queries, keys and values of proteins are calculated by the following formulas:

9 $$\begin{aligned} \left. \left\{ \begin{aligned}Q_P^i&=F_P\cdot W_q^i\\K_P^i&=F_D\cdot W_k^i\\V_P^i&=F_D\cdot W_v^i\end{aligned}\right. \right. , \end{aligned}$$

where the weight matrices $W_q^i\in \mathbb {R}^{D{_e}\times d_\text {head}}$, $W_k^i\in \mathbb {R}^{D{_e}\times d_\text {head}}$ and $W_v^i\in \mathbb {R}^{D{_e}\times d_\text {head}}$ share the same weights as drug attention. Through the application of a softmax function, the drug and protein attention matrices are computed as:

10 $$\begin{aligned}{} & {} A_D^i=\textrm{Softmax}\left( \frac{Q_D^i\cdot K_D^i {^\top }}{\sqrt{d_{K_D^i}}}\right) , \end{aligned}$$

11 $$\begin{aligned}{} & {} A_P^i=\textrm{Softmax}\left( \frac{Q_P^i\cdot K_P^i {^\top }}{\sqrt{d_{K_P^i}}}\right) , \end{aligned}$$

where $d_{K_D^i}=d_{K_P^i}=d_{head}$ is the dimension of *K* for drug and protein. The drug/protein feature map for each head is obtained by multiplying the drug/protein attention matrix of each attention head with the corresponding drug/protein value matrix. Subsequently, the drug/protein feature maps of all attention heads are concatenated in the channel dimension and fed into the linear layer to obtain the final drug feature representation $Z_P\in \mathbb {R}^{l_p\times D_p}$ and protein feature map $Z_D\in \mathbb {R}^{m_d\times D_d}$ received attention:

12 $$\begin{aligned}{} & {} Z_D=\text {Concat}(A_D^i\times V_D^i)\times W_Z, \end{aligned}$$

13 $$\begin{aligned}{} & {} Z_P=\text {Concat}(A_P^i\times V_P^i)\times W_Z, \end{aligned}$$

where $i=1,2,\cdots ,heads$ and $W_Z\in \mathbb {R}^{D_e \times D_e}$ is the shared weight matrix.

Next, the feature maps of interest are combined with the original feature maps to obtain the final drug feature map $F_{ZD}\in \mathbb {R}^{m_d\times D_d}$ and protein feature map $F_{ZP}\in \mathbb {R}^{l_p\times D_p}$:

14 $$\begin{aligned}{} & {} F_{ZD}=0.5Z_D+0.5F_D, \end{aligned}$$

15 $$\begin{aligned}{} & {} F_{ZP}=0.5Z_P+0.5F_P, \end{aligned}$$

The drug and protein feature maps are downsampled by using a global max-pooling operation to generate one-dimensional drug feature vector $d_{mp}\in \mathbb {R}^{D_d}$ and protein feature vector $p_{mp}\in \mathbb {R}^{D_p}$:

16 $$\begin{aligned}{} & {} d_{mp}=\text {Maxpooling}(F_{ZD}), \end{aligned}$$

17 $$\begin{aligned}{} & {} p_{mp}=\text {Maxpooling}(F_{ZP}), \end{aligned}$$

Finally, we concatenate $d_{mp}$ and $p_{mp}$ to obtain the joint feature representation $f\in \mathbb {R}^{2D_e}$:

18 $$\begin{aligned} f=\textrm{Concat}(d_{mp},p_{mp}), \end{aligned}$$

### Drug-target interaction prediction

In order to predict the DTI probability, we input the joint representation *f* into the decoder, which consists of a fully connected classification layer. Finally, the DTI probability *p* is generated as follows:

19 $$\begin{aligned} p=\sigma \left( Wf+b\right) , \end{aligned}$$

where *W* and *b* are learnable weight matrix and bias vector.

During model training, we employ backpropagation to concurrently optimize the learnable parameters. Our objective in training is to minimize the cross-entropy loss function:

20 $$\begin{aligned} \mathcal {L}=-\sum _i\left( y_i\log {(p_i)}+(1-y_i)\log {(1-p_i)}\right) +\frac{1}{2}\lambda ||\theta ||_2^2, \end{aligned}$$

where $y_i$ denotes the ground-truth label of the *i*-th drug-target pair. $p_i$ represents DTI prediction score predicted by the model. $\theta$ is the set of learnable weight matrices and bias vectors and $\lambda$ is a hyperparameter for L2 regularization to prevent overfitting.

### Cross-domain adaptation enhances generalization

**Fig. 2:**
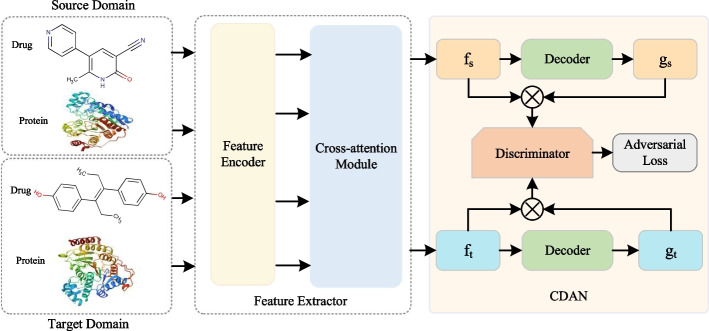
Diagram of cross-domain adaptation process. CDAN is a domain adaptation technique designed to address domain shift challenges with different distributions. We utilize CDAN to integrate the joint representation *f* of the source and target domain, along with classifier prediction *g* into the joint conditional representation distinguished by the discriminator. The discriminator is structured as a three-layer fully connected network with the specific goal of distinguishing the target domain from source domain by minimizing domain classification error

Deep learning models show excellent performance on similar data (i.e., in-domain) that is distributed with the training data. However, the performance on different data with different distributions (i.e., cross-domain) is not satisfactory. To this end, we employ  the CDAN module to improve the generalization ability of CAT-DTI model from a source domain rich in labeled data to a target domain containing only unlabeled data. Figure 2 shows the framework after integrating the CDAN module into CAT-DTI (i.e., ${\text {CAT-DTI}}_{\textrm{CDAN}}$), which consists of three key components: Feature Extractor $F\left( *\right)$, Decoder $G\left( *\right)$ and Discriminator $D\left( *\right)$.

On the cross-domain task, given $N_S$ labeled drug-target pairs $P_S=\{(x_s^i,y_s^i)\}_{N_S}^{i=1}$ in the source domain and $N_T$ unlabeled drug-target pairs $P_t=\{(x_t^i)\}_{N_T}^{i=1}$ in the target domain. We rely on CDAN to adjust the distribution of samples to optimize cross-domain prediction performance. The feature extractor $F\left( *\right)$ is the drug and protein feature encoder together with the cross-attention module to generate a joint representation of the input domain data, namely $f_s^i=F(x_s^i)$ and $f_t^j=F(x_t^j)$. For the decoder $G\left( *\right)$, we employ a fully connected classification layer and follow a softmax function as $G\left( *\right)$ to obtain predicted classification results $g_s^i=G(f_s^i)\in \mathbb {R}^2$ and $g_t^j=G(f_t^j)\in \mathbb {R}^2$. Subsequently, the joint representation *f* and the classifier prediction *g* are embedded into a joint conditional representation $c\in \mathbb {R}^{2D_e}$, which is defined as follows:

21 $$\begin{aligned} c=\text {FLATTEN}(f\otimes g), \end{aligned}$$

where $\text {FLATTEN}$ performs a flattening operation on the outer product of the *f* and *g* vectors and $\otimes$ is the outer product.

Adhering to CDAN principles, we employ a domain discriminator $D\left( *\right)$ to align the joint representation *f* and predicted classification distribution *g* of the source and target domains. $D\left( *\right)$ is a domain discriminator composed of a three-layer FCN that learns to distinguish whether a joint conditional representation *c* originates from the source or target domain. $F\left( *\right)$ and $G\left( *\right)$ are trained to minimize the cross-entropy loss $\mathcal {L}$ of the source domain with source label information, generating a joint conditional representation *c* that confuses the discriminator $D\left( *\right)$. In the cross-domain task, we utilize two losses: one for optimizing classification prediction and the other for optimizing the distribution alignment of the source and target domain:

22 $$\begin{aligned}{} & {} \mathcal {L}_S(F,G)=\mathbb {E}_{(x_s^i,y_s^i)\thicksim P_s}\mathcal {L}\left( G\left( F\left( x_s^i\right) \right) ,y_s^i\right) , \end{aligned}$$

23 $$\begin{aligned}{} & {} \begin{aligned} \mathcal {L}_{ad\nu }(F,G,D)&=\mathbb {E}_{x_t^i\thicksim P_t}\log\left( 1-D\left( f_t^i,g_t^i\right) \right) +\mathbb {E}_{x_s^j\thicksim P_s}\log(D(f_{s}^j,g_s^j)), \end{aligned} \end{aligned}$$

where $\mathcal {L}_S$ is the cross-entropy loss on the labeled source domain and $\mathcal {L}_{ad\nu }$ is the adversarial loss for the domain discriminator.

The optimization problem is written as a minimax paradigm:

24 $$\begin{aligned} \max _{\begin{array}{c}D\end{array}}\min _{\begin{array}{c}F,G\end{array}}\mathcal {L}_S(F,G)-\omega \mathcal {L}_{ad\nu }(F,G,D), \end{aligned}$$

where $\omega$ is a hyper parameter for weighting $\mathcal {L}_{ad\nu }$. By introducing adversarial training in $\mathcal {L}_{ad\nu }$, the difference in data distribution between the source domain and target domain is reduced, thereby enhancing the generalization ability of cross-domain prediction.

## Experiments and results

### Datasets and data processing

We comprehensively evaluate CAT-DTI and six baseline models on three public datasets: BindingDB, BioSNAP and Human. The BindingDB database records the binding affinity information of small drug molecules and proteins that have been verified through experiments and mainly studies the interaction between drug-like molecules and proteins. In experiments, we use a low-bias version of the BindingDB dataset constructed by previous research [35]. The BioSNAP dataset is constructed according to previous research [30, 36] derived from the DrugBank database [37], including 4510 drugs and 2181 proteins. It is designed as a balanced dataset containing validated positive samples and an equal number of unseen negative samples. Drawing on the previous studies [19, 29], we also employ a balanced version of the Human dataset containing equal numbers of positive and negative samples. The statistics of the three datasets are shown in Table 1.

**Table 1:** Details of datasets used in this work

Dadaset	Drug	Protein	Association
BindingDB	14,643	2,623	49,199
BioSNAP	4,510	2,181	27,464
Human	2,726	2,001	6,728

In experiments, we use different splitting strategies on the datasets for in-domain and cross-domain tasks. For in-domain evaluation, each experimental dataset is randomly split into training, validation and test sets in a ratio of 7:1:2. For cross- domain tasks, the decision to exclude the Human dataset stems from its comparatively limited sample size. In order to ensure the model has robust performance in cross-domain scenarios, the datasets should have sufficient data volume and sample diversity, so our cross-domain performance evaluation focuses on the large-scale BindingDB and BioSNAP datasets. We utilize the datasets from the previous study [31], which adopts a clustering-based pair split strategy to build cross-domain scenario and cluster drugs and target proteins from BindingDB and BioSNAP datasets respectively for cross-domain performance evaluation. Specifically, a single-linkage clustering method is used to cluster from the bottom and hierarchically to ensure that the distance between samples in different clusters always exceeds a predefined minimum distance threshold, which helps prevent the formation of clusters that are too close. For each dataset, the single-linkage algorithm is used for the clustering of drugs and proteins based on the ECFP4 [17] fingerprint and pseudo-amino acid composition (PSC) [38], respectively. Since the clustering-based pair split enables the quantitative construction of cross-domain task by taking into account the similarity between drugs and proteins, we use Jaccard distance and cosine distance on ECFP4 and PSC respectively to accurately measure pairwise distances. During the clustering of drugs and proteins, the distance threshold is set to 0.5 to ensure that the clusters do not become too large while maximizing the separation of different samples. So far, 2,780 drug clusters and 1,693 protein clusters have been obtained in the BindingDB dataset and 2,387 drug clusters and 1978 protein clusters have been obtained in the BioSNAP dataset. Through the clustering-based pair split strategy, the source and target domain are characterized by non-overlapping sets with different distributions. Following the general setup of domain adaptation, we use all labeled source domain data and 80% unlabeled target domain data as the training set and the remaining 20% labeled target domain data as the test set. While cross-domain evaluation presents greater challenges compared to in-domain random splitting, it emerges as a more efficacious methodology for assessing the generalization capacity of model in the practical realm of drug discovery.

### Baselines

We compare CAT-DTI with the following baselines.

- SVM [10] and RF [11] are used as a classifier to classify encoded drug and protein features.
- GraphDTA [23] uses GNN to encode drug molecule graphs and CNN to encode protein sequences. The learned drug and protein representation vectors are combined with a simple concatenation.
- TransformerCPI [29] treats drugs and proteins as two sequences, generating representations of protein sequences and drug atoms. The interaction feature is captured by Transformer decoder and the interaction probability is output by a fully connected layer.
- MolTrans [30] is a deep learning model that utilizes Transformer to encode drug and protein information and learns the interactions between substructures through a CNN-based interaction module.
- DrugBAN [31] encodes drug SMILES through GCN to obtain drug features and uses CNN to capture protein features. Drug-target pairwise interactions are captured by a bilinear attention network, followed by a fully-connected classifier for DTI prediction. Besides, conditional domain adversarial network is used to adjust the distribution of source and target domains in cross-domain prediction tasks.

### Evaluation metrics

Since DTI prediction is a classification task, we choose the widely recognized evaluation metrics AUROC (i.e., the area under the receiver operating characteristic curve) and AUPRC (i.e., the area under the precision-recall curve) as our primary assessment criteria. Additionally, we provide reports on Accuracy, Sensitivity, Specificity, and F1 score. In principally, the higher the AUROC value means the better the performance. The definitions of these evaluation metrics are given as:

25 $$\begin{aligned}{} & {} \text {Accuracy}=\frac{TP+TN}{TP+FP+TN+FN}, \end{aligned}$$

26 $$\begin{aligned}{} & {} \text {Sensitivity}=\frac{TP}{TP+FN}, \end{aligned}$$

27 $$\begin{aligned}{} & {} \text {Specificity}=\frac{TN}{TN+FP}, \end{aligned}$$

28 $$\begin{aligned}{} & {} F1=\frac{2TP}{2TP+FP+FN}, \end{aligned}$$

where the true positive (*TP*) and true negative (*TN*) are the number of drug-target with interaction and drug-target without interaction that are successfully identified, respectively. The false positive (*FP*) and false negative (*FN*) represent the number of drug-target with interaction and drug-target without interaction examples that are incorrectly identified.

### Experimental setting

During the experiments, we allow our model to run for up to 100 epochs on all datasets, setting the experimental batch size to 32. We use the Adam optimizer with a learning rate of $2.5\times 10^{-5}$ for the in-domain tasks and $5\times 10^{-5}$ for the cross-domain tasks. The maximum sequence length allowed for proteins is set to 1000 and the maximum number of atoms allowed for drug molecules is 290. The number of hidden neurons in the fully connected decoder is 512. Five independent experiments are run for each dataset split. The best performing model is the one that exhibits the highest AUROC on the validation set, which is used on the test set to get the performance metrics.

### Performance comparison

#### In-domain performance evaluation

In the in-domain scenario, we use regular CAT-DTI in our experiments, so we do not embed the CDAN into the model. We compare CAT-DTI with six baselines in the random split setting: SVM [10], RF [11], GraphDTA [23], TransformerCPI [29], MolTrans [30] and DrugBAN [31]. Table 2 shows the comparison on BindingDB, BioSNAP and Human datasets. The experimental results indicate that CAT-DTI demonstrates competitive performance across all evaluation metrics in the in-domain scenario. Specifically, CAT-DTI outperforms other comparative methods in terms of evaluation metrics such as AUROC, AUPRC, F1, and specificity on the BioSNAP and Human datasets, which is attributed to its strong capability in extracting key features of drugs and proteins, effectively capturing their interacting characteristics. It is noteworthy that CAT-DTI do not achieve the best performance on evaluation metrics such as AUROC, AUPRC, and F1 on the BindingDB dataset. One possible reason is that the key protein information in the BindingDB is mainly reflected in local features, which makes it challenging for the Transformer part of the protein feature encoder in CAT-DTI to fully utilize its potential, and indirectly affects the model’s ability to capture local features, leading to a decline in model performance. Nevertheless, CAT-DTI still outperforms other comparative methods in most cases.

**Table 2:** Comparison results of CAT-DTI and baselines on three datasets

Datasets	Methods	AUROC	AUPRC	F1	Sensitivity	Specificity	Accuracy
BindingDB	SVM [10]	0.904 ± 0.000	0.865 ± 0.001	0.785 ± 0.000	0.776 ± 0.000	0.857 ± 0.002	0.824 ± 0.001
	RF [11]	0.942 ± 0.001	0.923 ± 0.001	0.844 ± 0.002	0.840 ± 0.002	0.893 ± 0.002	0.871 ± 0.001
	GraphDTA [23]	0.944 ± 0.004	0.923 ± 0.006	0.880 ± 0.005	0.858 ± 0.026	0.897 ± 0.014	0.874 ± 0.010
	TransformerCPI [29]	0.947 ± 0.003	0.932 ± 0.004	0.888 ± 0.005	0.886 ± 0.016	0.890 ± 0.008	0.888 ± 0.007
	MolTrans [30]	0.947 ± 0.004	0.927 ± 0.006	0.886 ± 0.005	0.877 ± 0.018	0.894 ± 0.014	0.884 ± 0.007
	DrugBAN [31]	**0.961 ± 0.001**	**0.948 ± 0.001**	**0.903 ± 0.001**	**0.894 ± 0.011**	0.908 ± 0.009	**0.901 ± 0.003**
	CAT-DTI	0.960 ± 0.001	0.947 ± 0.001	0.900 ± 0.001	0.884 ± 0.010	**0.913 ± 0.009**	0.896 ± 0.002
BioSNAP	SVM [10]	0.819 ± 0.045	0.839 ± 0.038	0.827 ± 0.053	0.665 ± 0.046	0.835 ± 0.054	0.750 ± 0.050
	RF [11]	0.857 ± 0.001	0.872 ± 0.001	0.787 ± 0.001	0.763 ± 0.002	0.823 ± 0.001	0.793 ± 0.001
	GraphDTA [23]	0.871 ± 0.001	0.870 ± 0.005	0.807 ± 0.005	0.761 ± 0.015	0.838 ± 0.011	0.800 ± 0.005
	TransformerCPI [29]	0.876 ± 0.004	0.881 ± 0.007	0.803 ± 0.006	0.768 ± 0.024	0.827 ± 0.012	0.797 ± 0.008
	MolTrans [30]	0.895 ± 0.006	0.899 ± 0.006	0.825 ± 0.007	0.791 ± 0.032	0.848 ± 0.014	0.820 ± 0.011
	DrugBAN [31]	0.902 ± 0.001	0.905 ± 0.002	0.838 ± 0.003	**0.825 ± 0.014**	0.847 ± 0.006	0.836 ± 0.004
	CAT-DTI	**0.909 ± 0.002**	**0.907 ± 0.004**	**0.840 ± 0.004**	0.816 ± 0.012	**0.857 ± 0.006**	**0.836 ± 0.005**
Human	SVM [10]	0.913 ± 0.000	0.905 ± 0.000	0.811 ± 0.000	0.782 ± 0.000	0.830 ± 0.000	0.838 ± 0.000
	RF [11]	0.939 ± 0.002	0.927 ± 0.001	0.848 ± 0.005	0.833 ± 0.006	0.893 ± 0.007	0.866 ± 0.006
	GraphDTA [23]	0.965 ± 0.003	0.955 ± 0.003	0.907 ± 0.008	0.912 ± 0.017	0.904 ± 0.016	0.908 ± 0.008
	TransformerCPI [29]	0.954 ± 0.002	0.941 ± 0.002	0.891 ± 0.005	0.831 ± 0.023	0.939 ± 0.018	0.879 ± 0.007
	MolTrans[30]	0.981 ± 0.002	0.976 ± 0.002	0.943 ± 0.005	**0.949 ± 0.011**	0.939 ± 0.017	0.941 ± 0.004
	DrugBAN [31]	0.981 ± 0.001	0.969 ± 0.005	0.940 ± 0.004	0.938 ± 0.010	0.941 ± 0.013	0.940 ± 0.003
	CAT-DTI	**0.983 ± 0.001**	**0.976 ± 0.003**	**0.944 ± 0.002**	0.929 ± 0.007	**0.957 ± 0.008**	**0.942 ± 0.002**

Bold values indicate the best results achieved by all these competitive methods

Overall, the experimental results on the three datasets all confirm the effectiveness of the CAT-DTI for DTI prediction. The improvement of experimental results is attributed to our method not only capturing protein local features but also analyzing global context information for protein features. Moreover, the incorporation of the cross-attention module enables the model to concurrently consider the impact of drug features on proteins and the influence of protein features on drugs. This bidirectional interaction empowers CAT-DTI to comprehensively comprehend and capture the intricate interactions between drugs and proteins, thereby achieving the effective fusion of drug and protein target features.

#### Cross-domain performance evaluation

In-domain classification tasks under random split are relatively simple and of limited practical value. In order to better simulate real-world situations, we focus on the more challenging cross-domain DTI prediction, where the training data and test data have different distribution characteristics. In order to deeply explore the knowledge transferability in cross-domain prediction, we embed the CDAN module into the CAT-DTI model, which means using CAT-DTI$_{\text {CDAN}}$ for cross-domain prediction.

We present the cross-domain performance evaluation results on BindingDB and BioSNAP datasets in Table 3. All methods show a significant drop compared to the previous in-domain prediction results due to the reduced information overlap between training and test datasets. However, our newly proposed CAT-DTI model clearly outperforms other state-of-the-art models on both datasets. Specifically, the AUROC and AUPRC of CAT-DTI on the BioSNAP dataset are 10.4% and 7.1% higher than the second-ranked DrugBAN. At the same time, the AUROC and AUPRC of CAT-DTI on the BindingDB dataset are 12.4% and 15.4% higher than those of DrugBAN. What’s more worth mentioning is that even when the CDAN module is incorporated into DrugBAN (i.e., DrugBAN$_{\text {CDAN}}$), the performance of CAT-DTI outperforms DrugBAN$_{\text {CDAN}}$ model. Experimental results demonstrate that the proposed CAT-DTI can effectively handle cross-domain DTI prediction problems. Compared with previous methods, CAT-DTI not only exhibits improved accuracy but also demonstrates strong cross-domain generalization capability. We attribute the superiority of CAT-DTI in cross-domain generalization to its unique framework design and the collaborative interaction of key components. The introduced cross-attention module enables CAT-DTI to simultaneously consider drug and protein features, establishing bidirectional information correlation and aiding in a more comprehensive and accurate capture of interactions across different domains. This feature fusion method enhances the adaptability of CAT-DTI to diverse data distributions, thereby improving cross-domain generalization performance. Additionally, the protein feature encoder combines CNN and Transformer to provide CAT-DTI with comprehensive modeling capability for local and global information within protein sequences.

**Table 3:** Cross-domain performance comparison of CAT-DTI and other baselines on BindingDB and BioSNAP Datasets

Dataset	Method	AUROC	AUPRC	F1	Accuracy
BindingDB	SVM [10]	0.490 ± 0.015	0.460 ± 0.001	0.162 ± 0.158	0.531 ± 0.009
	RF [11]	0.493 ± 0.021	0.468 ± 0.023	0.109 ± 0.029	0.535 ± 0.012
	GraphDTA [23]	0.536 ± 0.015	0.496 ± 0.029	0.668 ± 0.001	0.472 ± 0.009
	TransformerCPI [29]	0.597 ± 0.041	0.562 ± 0.031	0.670 ± 0.005	0.490 ± 0.027
	MolTrans [30]	0.554 ± 0.024	0.511 ± 0.025	0.668 ± 0.001	0.470 ± 0.004
	DrugBAN [31]	0.576 ± 0.023	0.535 ± 0.014	0.668 ± 0.002	0.471 ± 0.012
	DrugBAN$_{\text {CDAN}}$ [31]	0.604 ± 0.027	0.570 ± 0.047	0.675 ± 0.004	0.509 ± 0.021
	CAT-DTI	0.636 ± 0.013	0.573 ± 0.020	0.688 ± 0.004	0.553 ± 0.024
	CAT-DTI$_{\text {CDAN}}$	**0.678 ± 0.005**	**0.626 ± 0.021**	**0.690 ± 0.004**	**0.572 ± 0.016**
BioSNAP	SVM [10]	0.602 ± 0.005	0.528 ± 0.005	0.400 ± 0.122	0.513 ± 0.011
	RF [11]	0.590 ± 0.015	0.568 ± 0.018	0.018 ± 0.010	0.499 ± 0.004
	GraphDTA [23]	0.618 ± 0.005	0.618 ± 0.008	0.672 ± 0.003	0.535 ± 0.024
	TransformerCPI [29]	0.645 ± 0.022	0.642 ± 0.032	0.681 ± 0.009	0.558 ± 0.025
	MolTrans [30]	0.621 ± 0.015	0.608 ± 0.022	0.675 ± 0.006	0.546 ± 0.032
	DrugBAN [31]	0.630 ± 0.007	0.622 ± 0.018	0.671 ± 0.004	0.537 ± 0.034
	DrugBAN$_{\text {CDAN}}$ [31]	0.685 ± 0.044	0.713 ± 0.041	0.677 ± 0.010	0.565 ± 0.056
	CAT-DTI	0.708 ± 0.008	0.718 ± 0.009	0.695 ± 0.008	0.618 ± 0.031
	CAT-DTI$_{\text {CDAN}}$	**0.729 ± 0.010**	**0.733 ± 0.016**	**0.699 ± 0.008**	**0.633 ± 0.021**

Bold values indicate the best results achieved by all these competitive methods

**Fig. 3:**
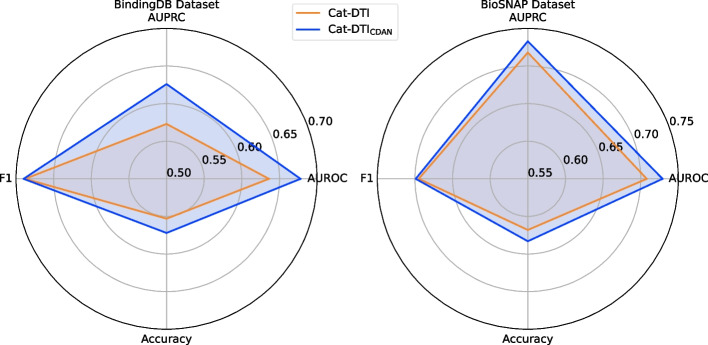
Cross-domain performance comparison of CAT-DTI with and without CDAN module on BindingDB and BioSNAP datasets

In recent years, domain adaptation techniques have attracted extensive attention in academia due to their excellent cross-domain knowledge transfer capabilities. In our work, we explore and improve cross-domain DTI prediction by combining CAT-DTI model with CDAN. As shown in Table 3, the proposed CAT-DTI$_{\text {CDAN}}$ model has a significant performance improvement after integrating the domain adaptation module. Specifically, compared with the CAT-DTI model, CAT-DTI$_{\text {CDAN}}$ improves AUROC and AUPRC on the BindingDB dataset by 6.6% and 9.2% and also has a gratifying improvement on the BioSNAP dataset. In order to more clearly observe the improvement effect of adding the domain adaptation module CDAN to CAT-DTI, we draw the prediction results of CAT-DTI and CAT-DTI$_{\text {CDAN}}$ on BindingDB and BioSNAP datasets as radar charts, as shown in Fig. 3. In this way, we can more intuitively observe the positive impact of the CDAN module on cross-domain tasks. By reducing cross-domain distribution bias, CAT-DTI demonstrates a substantial enhancement in its generalization performance with the incorporation of the CDAN module. These experimental results further confirm the significant superiority of CAT-DTI in cross-domain generalization ability.

We analyze that the cross-domain generalization performance of CAT-DTI$_{\text {CDAN}}$ has been improved is attributed to CAT-DTI$_{\text {CDAN}}$ can better optimize feature alignment and effectively reduce the distribution differences between different domains after introducing the CDAN module. Through the adversarial learning mechanism, CAT-DTI$_{\text {CDAN}}$ is able to adjust the feature representations of the source domain and the target domain to make them statistically more similar, thereby improving the model’s performance on the target domain. This domain adaptation mechanism enables CAT-DTI to better adapt to new and unseen domain data and enhances the generalization ability of the model. Specifically, the introduction of the CDAN module helps reduce domain differences and makes CAT-DTI more robust when processing new drug and protein interaction data, thus improving its prediction accuracy and adaptability.

### Ablation experiments

**Fig. 4:**
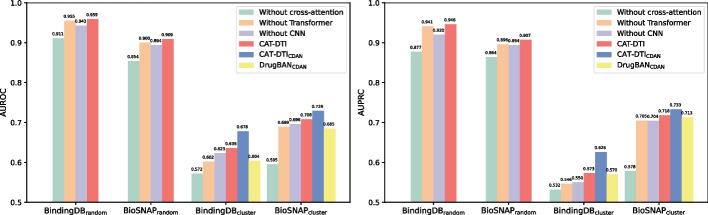
AUROC and AUPRC for random split and clustering-based split strategies on BindingDB and BioSNAP datasets

To explore the effect of CNN combined with Transformer for protein feature extraction, DTI feature captured by cross-attention module and domain adaptation module CDAN on model prediction performance, we perform ablation experiments. In the in-domain task, we compare the experimental results of CAT-DTI with three variant models, including CAT-DTI using only CNN for protein feature encoder after removing the Transformer (i.e., Without Transformer), the model using only Transformer for protein feature encoder after removing the CNN (i.e., Without CNN) and the model removing the cross-attention module (i.e., Without cross-attention). In the cross-domain task, in order to evaluate the efficacy of CDAN, we compare the evaluation results of CAT-DTI$_{\text {CDAN}}$ with CDAN module removed (i.e., CAT-DTI) and DrugBAN fused with CDAN (i.e., DrugBAN$_{\text {CDAN}}$). The experimental results are illustrated in Fig. 4.

By analyzing the experimental results, we observed an enhancement in the predictive performance of CAT-DTI when the model integrated the complete module, which confirms the effectiveness of the mechanism that combines CNN and Transformer for protein feature extraction, highlights the efficient interactive capabilities of the cross-attention module, and indicates the improvement in cross-domain performance of CAT-DTI with the introduction of the CDAN module.

### Case study

In order to further verify the effectiveness of CAT-DTI, we selected two representative targets for case study, namely P00519 (Tyrosine-protein kinase ABL1) and P35228 (Nitric oxide synthase, inducible). Based on the testing result from the test set of BindingDB, we select drug-target pairs containing P00519 and P35228, and then rank the candidate drugs for each protein in descending order according to their predicted interaction scores, choosing the top 6 for each target as depicted in Table 4. After detailed review, the drug and target pairs listed in the table have been confirmed to have interactions in the Drugbank database. Taking the example of the drug-target pair involving the target protein P00519 and its first candidate drug DB08901 (Ponatinib) from Table 4, the tyrosine-protein kinase ABL1 plays a pivotal role in various processes related to cell growth and survival. It coordinates actin cytoskeleton dynamics by regulating protein tyrosine phosphorylation. On the other hand, DB08901 (Ponatinib) is a novel Bcr-Abl tyrosine kinase inhibitor that has been proven to inhibit the tyrosine kinase activity of Abl and is used to treat chronic myelogenous leukemia. Therefore, the drug numbered DB08901 has been verified as a ligand for the target protein numbered P00519.

**Table 4:** Top-ranked list of predicted drugs for two proteins

Protein	Drug	Prediction score
P00519 (Tyrosine-protein kinase ABL1)	DB08901	1.0000
	DB08896	0.9967
	DB01254	0.9831
	DB08350	0.9520
	DB12267	0.9438
	DB08043	0.9118
P35228 (Nitric oxide synthase, inducible)	DB07002	0.9930
	DB02044	0.9912
	DB07318	0.9862
	DB07405	0.9810
	DB09237	0.9444
	DB07388	0.9293

**Table 5:** DTI prediction results for five drugs

Drug	Protein	Prediction score
DB00786 (Marimastat)	P51512	0.9983
	P08253	0.9935
	P39900	0.9703
DB01254 (Dasatinib)	P12931	0.9862
	P00519	0.8722
DB06155 (Rimonabant)	P21554	0.9990
DB00482 (Celecoxib)	P35354	0.9980
DB00481 (Raloxifene)	Q92731	0.9359

Furthermore, we have selected additional 5 drugs to expand our case study, including DB00786 (Marimastat), DB01254 (Dasatinib), DB06155 (Rimonabant), DB00482 (Celecoxib), and DB00481 (Raloxifene). Based on the testing results, we once again selected drug-target pairs from the test set of BindingDB dataset that contain the aforementioned drugs and have higher predicted interaction scores, as shown in Table 5. Similarly, all drug-target pairs in Table 5 can be found in the DrugBank database, indicating that evidence of interactions for these drug-target pairs can be found in the DrugBank database. For example, DB01254 (Dasatinib) is a tyrosine kinase inhibitor that can inhibit the activity of P00519 (Tyrosine-protein kinase ABL1) and P12931 (Proto-oncogene tyrosine-protein kinase Src).

The above cases demonstrate that our proposed CAT-DTI can effectively predict potential drug-target pairs, possessing the capability to identify potential candidate drugs and thus improving the virtual screening stage of drug discovery.

## Conclusion

In this work, we propose a deep learning model named CAT-DTI, which is based on cross-attention and Transformer to enhance the accuracy of predicting drug-target interactions. We employ GCN for extracting drug features, while the acquisition of protein target features uses CNN combined with Transformer, which can not only capture local features of proteins, but also take into account global context information. The introduction of the cross-attention module effectively facilitated bidirectional feature interactions between drugs and proteins, leading to the extraction of more critical DTI features. Furthermore, with the help of CDAN, our model exhibits good adaptability and predictive performance in cross-domain task, which enhances the generalization performance of CAT-DTI. Compared with other state-of-the-art models and traditional machine learning models, experimental results show that CAT-DTI improves DTI prediction performance in both in-domain and cross-domain setting, especially making promising progress in cross-domain prediction tasks.

## Data Availability

The data and code can be found online at: https://github.com/ZXT0212/CAT-DTI.
